# Sublethal concentrations of conventional nematicides alter the physiological activities of *Meloidogyne incognita* and suppress parasitism

**DOI:** 10.1038/s41598-022-27270-z

**Published:** 2023-01-05

**Authors:** Abdullah A. Abdel-Rahman, Hosny H. Kesba, Hoda G. Mohamed, Donia F. Kamel, Fatma S. Ahmed

**Affiliations:** 1 Zoology and Agricultural Nematology Department, Faculty of Agriculture, Cairo University, Giza, 12613 Egypt; 2 Faculty of Agriculture, Cairo University, Giza, 12613 Egypt; 3 Department of Economic Entomology and Pesticides, Faculty of Agriculture, Cairo University, Giza, 12613 Egypt

**Keywords:** Biochemistry, Neuroscience, Zoology

## Abstract

Reducing nematicide dose rates could be a useful strategy for mitigating their negative effects on health and the environment. In this study, enzymatic activities and the parasitic ability of *Meloidogyne incognita* after exposure to sub-lethal concentrations (0.25, 1, 2, and 5 ppm) of ethoprophos, fenamiphos, and oxamyl were investigated. Although the tested concentrations did not show nematicidal properties in vitro, they reduced root galls by at least 30% at 0.25 ppm and up to 67% at 5 ppm in pots, besides disrupting nematode fertility. For all three nematicides at 2 ppm, a chemotaxis assay showed that ≤ 11% of the nematode population was successfully oriented to the host roots, compared to 44% in the control. Ethoprophos and fenamiphos at 5 ppm showed poor inhibitory effects on acetylcholinesterase (AChE) activity (5.6% and 12.5%, respectively). In contrast, the same nematicides were shown to be strong ATPase inhibitors, causing 82.4% and 82.8% inhibition, respectively. At the same concentration, oxamyl moderately inhibited AChE and ATPase-specific activities, the inhibition being 22.5% and 35.2%, respectively. This study suggests that the use of very low nematicide concentrations could be a promising strategy for nematode management. Furthermore, it has also highlighted the role of ATPases as a possible target site for suppressing nematode activity in the development of future nematicides.

## Introduction

Plant–parasitic nematodes are one of the most damaging plant pests^1^. Worldwide annual yield losses due to nematode infection are estimated to be approximately US$157 billion^2^. Among several plant parasitic nematodes, root-knot nematodes (*Meloidogyne* spp.) have an extremely wide host range^3,4^, and are responsible for massive crop losses in okra^5^, cotton^6^, tomato^7^, eggplant^8^, cucumber^9^, sweet potato^10^, and black pepper^11^. This made this pest one of the most threatening polyphagous endoparasites found all over the world^12^.

To manage *Meloidogyne* spp, a variety of nematicides are used. However, the widespread use of these nematicides may have negative consequences for human health, beneficial organisms, and the environment^13^. Thus, using nematicides to manage this pest with a minimal negative impact on the ecosystem may be a positive option. It is imperative to ask whether low levels of nematicides can similarly suppress nematodes as the higher doses do. Concerning this, some researchers have previously reported the potential effects of sublethal nematicide concentrations on the management of nematodes. For example, fluazaindolizine at 5 ppm was found to reduce the *Meloidogyne incognita* galls and eggs^14^, and fluazaindolizine at 5–10 ppm inhibited *Heterodera schatii* development inside the roots of *Arabidopsis thaliana*, without effects on mobility, host recognition, penetration, or the establishment of feeding sites^15^. The ED50 (effective doses that caused inactivity for 50% of the population) values for oxamyl, fluensulfone, and fluazaindolizine (180, 131, and 89 ppm respectively) were found to suppress *M. incognita* reproduction in tomatoes^16^. At LC_20_, emamectin benzoate, ivermectin, and milbemycin showed negative effects on the embryonic stages, reduced nematode larval size, development, body length and nematicidal effects on *Bursaphelenchus xylophilus*^17^*.*

The two major nematicide categories used to manage *M. incognita* are carbamates and organophosphates. Both groups target the nematode nervous system by suppressing the acetylcholinesterase (AChE) enzyme^18^. Although the general symptoms of acetylcholinesterase deactivation in nematodes include paralysis after hyper-activity actions, some studies have reported behavioral changes in nematodes as a result of exposure to AChE inhibitors at lower concentrations than those required to cause paralysis^19^. Many studies have also reported that carbamates and organophosphates nematicides inhibit AChE levels, making AChE an important biomarker of these nematicides exposure.

ATPases, are one of the most common clusters of enzymes in nematodes, are also biomarkers that could affect nematode viability^20^. These enzymes are involved in many physiological processes in nematodes, including ionic gradient management, muscle contractions, cuticle synthesis, nutrition, osmoregulation, detoxification, and reproduction^21^. As a result of their diverse functions, ATPases have been proposed as potential target sites for nematicides^22^^–^^24^. Despite this, there is a paucity of information in the literature about the impact of nematicides on these ATPases.

The main objective of the current study is to evaluate the impact of extremely low nematicide concentrations on *M. incognita* AChE and ATPase activities, infection, and reproduction. It is hypothesized that the low concentrations of the conventional chemicals will alter the physiological fitness of *M. incognita*, influencing nematode behavior, and thus has a deleterious impact on parasitism and fecundity.

## Materials and methods

The efficacy of low-concentration (0.25, 1, 2, and 5 ppm) applications of three carbamate and OP nematicides against the *Meloidogyne incognita* in comparison with high concentrations (250 ppm), were investigated using in vitro experiments. The changes associated with the nematode acetylcholinesterase and ATPase enzymes and the effects on nematode-host recognition, infection, and reproduction in pots were also assessed.

### *Meloidogyne incognita* inoculum source and juveniles’ collection

A pure stock culture of the *M. incognita* (Chitwood, 1949) was established and maintained on eggplant for three months in pots with a 20 cm diameter containing 1:1 sandy clay soil. The egg masses of *M. incognita* were collected from heavily infected roots. The egg masses were then rinsed with distilled water and incubated in a Baermann unit above double-layered tissue paper^25^. The hatched juveniles were collected every 24–48 h under room temperature conditions (28 ± 2 °C), and then concentrated and used for laboratory and pot experiments.

### Pesticides and test concentrations

Formulated oxamyl (Mastot® 24SL), ethoprophos (Kafrophos Extra® 40EC), and fenamiphos (Fenamor® 40EC) were purchased from local agricultural chemical stores and kept in a refrigerator (4 ± 2 °C). The concentrations used were: oxamyl at 0.25, 1, 2, and 5 mg/L, and these were 576-, 144-, 72-, and 29-fold lower than the field recommended concentrations (3 L/feddan (feddan = 4200 m^2^); drench application consumes ≈ 5000 L/ fed., equal to 144 mg a.i./L), respectively; ethoprophos and fenamiphos at 0.5, 1, 2, and 5 mg/L, which were 640-, 320-, 160-, and 64-fold lower than the field use concentrations (4 L/feddan, equal to 320 mg a.i./L), respectively. For enzyme determination, concentrations of 5 and 250 mg/L oxamyl were prepared, but the ethoprophos and fenamiphos concentrations were 8 and 400 mg/L.

### In vitro survival tests

Approximately 200 M*. incognita* juveniles were tested for survival after 72 h exposure to concentrations of 0.25, 1, 2, 5, 250 mg/L a.i. of ethoprophos, fenamiphos, and oxamyl. Five replicates for each treatment were prepared in test tubes and kept at room temperature (28 ± 2 °C) conditions. The control group included five replicates of 200 juveniles in only tap water. Mobile and immobile nematode juveniles were counted using the light binocular microscope (100X magnification). Corrected-mortality percentages were calculated according to the Sun-Shepard formula^26^ for non-uniform populations as follows:

$$\mathrm{Corrected\,mortality }(\mathrm{\%})=\frac{\mathrm{Mortality\,\%\,in\,treated\,plot }\pm \mathrm{Change\,\%\,in\,control\,plot\,population}}{100\pm \mathrm{Change\,\%\,in\,control\,plot\,population}}\times 100$$

### Nematode assay

All eggplant experiments were carried out following the relevant guidelines and regulations approved for the use of plants by Cairo University's Faculty of Agriculture. The vice dean for environmental affairs and community services approved the transplanting and collection of eggplants for research purposes from Cairo University's Faculty of Agriculture greenhouses.

Three-week-old eggplant seedlings were transplanted (one seedling per pot) and after acclimatization, each pot was inoculated with approximately 2000 *M. incognita* second-stage juveniles (J2). Inoculation was conducted by dispensing the nematode suspension around the seedling’s roots after removing the upper soil layer, and there were three replicates for each treatment. Then, 45 days after inoculation, the plants were removed from the pots and each root system was soaked and separated. The galls of the *M. incognita* and the number of egg masses were examined and counted under a stereo dissecting microscope (20× magnification). The number of eggs per egg mass was obtained by calculating the mean after collecting 15 random full egg masses from each treatment. The pot experiment was repeated twice during the summer season. Data of eggs per eggmass were not statistically analyzed as the numbers of eggs/egg mass is the mean of the whole treatment.

### Host identification pipette-tube assay

**Figure 1:**
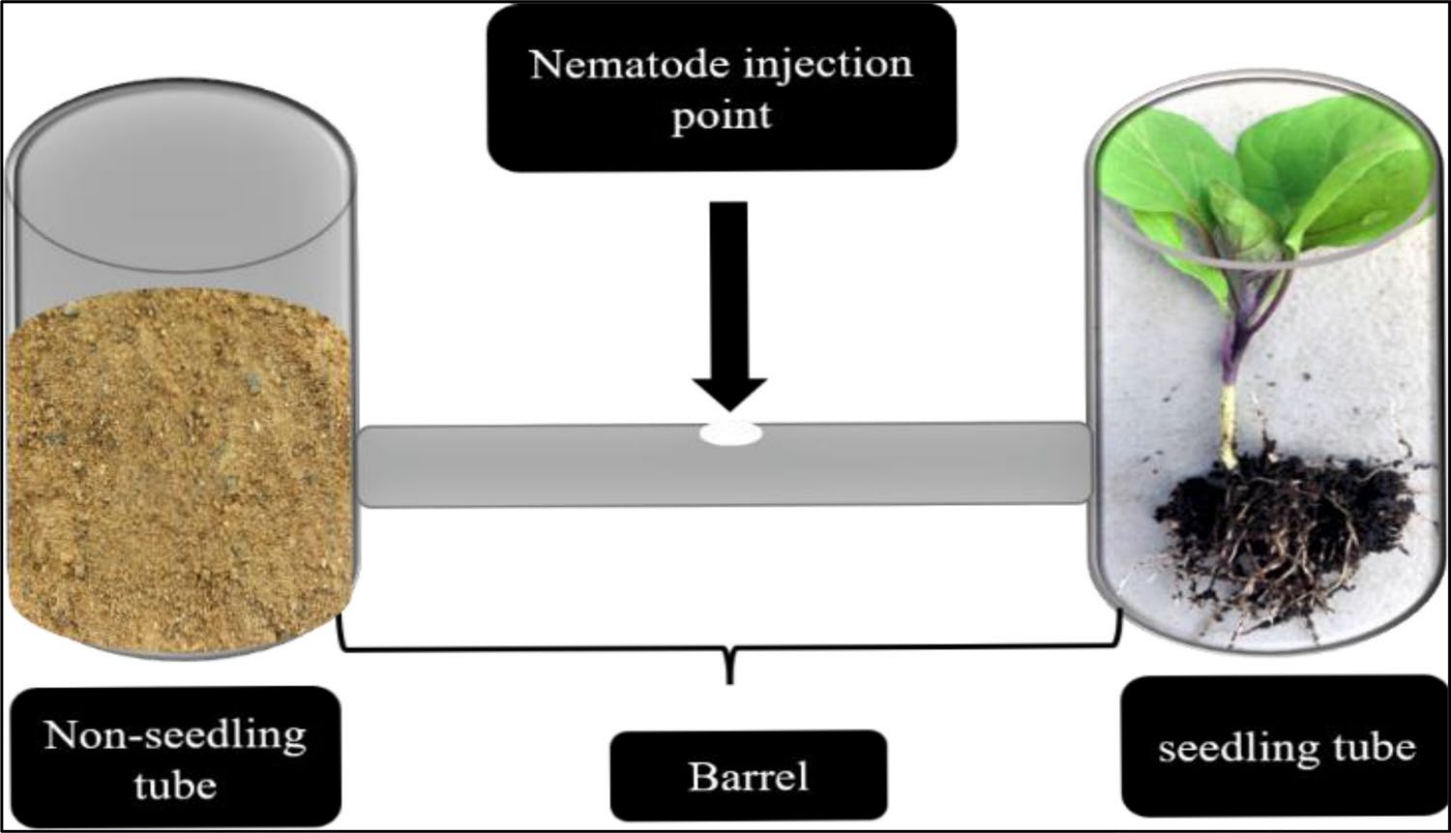
Schematic diagram showing the host finding pipette-tube assay unit (chemotaxis assay).

As shown in (Fig. 1), two 50 mL plastic falcon tubes (2.5 cm diameter, 10 cm long) were cut open 1 cm from the tube terminus and the other terminus was kept closed with the tube lid according to^27^ with a slight modification; one circular incision (1.5 cm diameter) in each wide tube was made 1 cm from the bottom. A narrower plastic tube (1.5 cm diameter, 5 cm long) was used as a horizontal bridge between the two wide vertical tubes, and the circular incisions were the connection points between the narrower middle tube and the two wider tubes. Removable glue was used to fasten the tubes together, and ultimately an H-shaped hollow unit was formed. The middle connecting tube is called ‘the central barrel’, one of the wide tubes contained a seedling of a susceptible host (4–5-day Zucchini seedlings) and this called a ‘seedling bulb’, and the other wide tube contained only sand and this called non-seedling bulb. A central pore called an ‘injection point’ was made in the middle of each central barrel and 0.5 mL of the nematode suspension containing approximately 800 s-stage Juveniles (J2) of *M. incognita* was injected. Humid conditions were maintained inside the units by adding 1–2 mL of tap water daily to each wide tube. After 72 h, the sand inside each part (seedling, non-seedling tube, and middle barrel) was collected, and nematode juveniles were extracted using the sieve method and counted using a light microscope (100X magnification). The host identification pipette-tube assay was repeated twice.

### Enzyme activity assays

#### Chemicals

The following chemicals were purchased from Sigma-Aldrich (Sigma- Aldrich, St. Louis, MO): acetylthiocholine iodide (ATChI), 5,5′ -dithiobis (2-nitrobenzoic acid) (DTNB), adenosine 5′-triphosphate (ATP), ammonium molybdate tetrahydrate, and trichloroacetic acid. All other chemicals used were of the highest grade commercially available.

#### Enzyme preparation

At least 95% nematode breakage should be obtained to accurately determine the enzyme activities. To achieve this, a small number of glass beads (≤ 106 μm Sigma-Aldrich) were added to a 1 mL nematode suspension containing approximately 10,000 juveniles, and it was homogenized for 30 s. To increase nematode cracking, the juveniles were then homogenized in 10 mL ice-cold 100 mM phosphate buffer (pH 7.0) to determine the acetylcholine esterase (AChE) activity or 50 mM Tris–HCl buffer (pH 7.4) to determine the adenosine triphosphatase (ATPase) total activity using Surom™ homogenizer 50–60 Hz in five periods, none of which exceeded 30 s. The enzyme extracts were then centrifuged at 5,000 rpm for 20 min at 0 °C using Sigma- 4K15C Centrifuge and used as an enzyme source to assay the AChE activity. To determine the total activity of the ATPase, the homogenates were centrifuged at 5,000 rpm for 10 min at − 4 °C, then the supernatant was centrifuged at 15,000 rpm for 30 min at − 4 °C. The pellets were resuspended in 50 mM Tris–HCl buffer (pH 7.4).

#### Acetylcholine esterase (AChE) activity assay

AChE activity was measured using ATChI according to the method of^28^ which was modified by^29^ with adjustments to the sample volume, ATChI, and DTNB volumes, and the period in which the absorbance (∆A) was read**.** Each reaction mixture included a 250 μL enzyme extract, 50 μL of 156 mM ATChI, and 1200 μL of 0.25 mM DTNB in 0.1 M phosphate buffer, pH 8.0. After allowing the reaction to stabilize for 60 s, the change in absorbance (∆A) was measured at 30-s intervals for 10 min. A Thermo scientific evolution 100 UV–Visible spectrophotometer was used to measure the ATChI hydrolysis at 405 nm at 25˚C. Three replicate measurements were carried out for each sample. AChE activity was normalized to the protein content and specific activity was expressed as nmol ATChI hydrolyzed per minute per-mg protein using the extinction coefficient of 1.36 × 10^4^ M^−1^ cm^−1^^28^.

#### Adenosine triphosphatase (ATPase) total activity assay

The ATPase enzyme activity was determined calorimetrically according to^30^, with adjustments made to the sample volume, incubation period, and the amount of phosphorus stain used. A 250 μL enzyme suspension was added to a 100 μL mixture of 100 mM NaCl and 20 mM KCl, 100 μL of 5 mM Mg_2_Cl, and 50 μL of 5 mM ATP, and the volume was adjusted to 850 μL with Tris–HCl buffer (pH 7.4). The mixture was then incubated at 37 °C for 15 min before being terminated with 150 μL of ice-cold trichloroacetic acid (TCA) at 20% (w/v). One milliliter of freshly prepared phosphorus reagent stain (10% ammonium molybdate solution prepared in 10 N sulfuric acid) was added to the mixture. A Thermo scientific evolution 100 UV–Visible spectrophotometer was used to measure the absorbance at 740 nm. Enzyme activity was represented as nmol P_i_/mg protein/15 min**.** In the enzymatic assays, the inhibition percentages for the activities were compared to those of the controls.

#### Protein assay

Total protein was determined according to^31^ using a Coomassie brilliant blue dye and bovine serum albumin as a standard. For each nematode extract, three 250 μL replicates were tested. After 5 min, the OD at 595 nm was measured against blanks and converted to the protein concentration (mg/mL) using the standard absorbance curve of known concentrations of bovine serum albumin. The protein content for each sample was used to standardize the AChE and ATPase-specific activity to a per-mg protein basis.

### Statistical analysis

Using SPSS software (Version 15.0, SPSS Inc., Chicago, IL, U.S.A., https://www.ibm.com/products/spss-statistics), the AChE and ATPase activity data were both analyzed using a one-way analysis of variance, followed by Duncan's multiple range test (*P* ≤ 0.05) to separate the means.

## Results

### In vitro effects of nematicides sub-lethal concentrations on *M. incognita* juveniles

**Figure 2:**
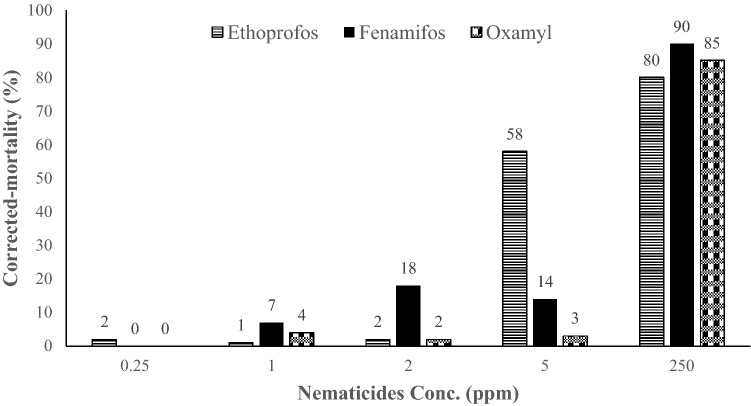
In vitro corrected- mortality of *Meloidogyne incognita* juveniles treated with different concentrations of the three chemical nematicides at 72 h post-treatment.

The mortality in vitro after 72 h was found to be associated with the different concentrations of ethoprophos, fenamiphos, and oxamyl (Fig. 2). The mortality percentage values of the ethoprophos and oxamyl treatments at 0.25, 1, and 2 ppm were found to be close to the values in the control and they generally did not exceed 10%; there were some slight fluctuations among the treatments that were not concentration-dependent. At 5 ppm, there was a sharp increase in the juvenile mortality for ethoprophos to 58%. The highest concentration (250 ppm) of ethoprophos, fenamiphos, and oxamyl resulted in the highest levels of mortality at 85, 90, and 80%, respectively.

### Effects of drench application of nematicides sub-lethal concentrations on *M. incognita* infection and fecundity in the pot experiment

The infectivity and fecundity of the *M. incognita* juveniles on the susceptible host (eggplant) after drench applications of the different sublethal and lethal concentrations of the three nematicides are shown in Table 1. Generally, all treatments caused significant reductions in gall and egg mass numbers when compared with the control. At lower concentrations of 0.25, 1, and 2 ppm, the fenamiphos and oxamyl applications resulted in the highest significant reductions in gall numbers, and there were no significant differences between these two nematicides. For ethoprophos, with the same mentioned concentrations, the lowest gall number reductions were achieved. At 5 and 250 ppm, the gall numbers were reduced to 309 and 126 with fenamiphos, and 275 and 179 with oxamyl, respectively, achieving the highest suppression of gall formation when compared to the control (832 gall). Ethoprophos was found to be phytotoxic at 250 ppm, resulting in a poor root fresh weight (0.3 g) when compared with the control (7.5 g). In contrast, the oxamyl drench application enhanced root growth at 5 and 250 ppm, as the root weight was reportedly 10.4 and 28.2 g, respectively. Efficiency (the percentage reduction in gall numbers over the control), reflected the previous situation for gall numbers more clearly, as all nematicides at concentrations of 0.25, 1, and 2 mg/L resulted in > 30% efficiency and increased to 54% and 59% when there was 2 ppm fenamiphos and oxamyl, respectively. In addition, 5 ppm fenamiphos and oxamyl resulted in efficiency values of 63% and 67%, which were slightly lower than the efficiency percentage values reported for the highest concentration of 250 ppm (85% and 79%, respectively).

**Table 1:** Effects of sublethal chemical nematicide concentrations on the infectivity and fecundity of *Meloidogyne incognita* on potted eggplant.

	Conc (ppm)	Root Wt	Galls	Eggmass	Eggs/egg mass	% Efficiency
Ethoprophos	0.25	6.7^d^	584^b^	302^ab^	256	30
	1	7.6^cd^	559^b^	137^c^	200	33
	2	4.3^f^	522^bc^	154^c^	200	37
	5	4.7^ef^	469^c^	243^b^	89	44
	250	0.3 g	–	–	–	Phytotoxicity
Fenamiphos	0.25	4.7^ef^	485^c^	40^d^	250	42
	1	6.1^de^	377^d^	316^ab^	176	55
	2	7.3^d^	382^d^	100^cd^	170	54
	5	6.0^de^	309^e^	44^d^	170	63
	250	7.3^cd^	126^f^	34^d^	90	85
Oxamyl	0.25	7.4^cd^	401^d^	49^d^	200	52
	1	7.5^cd^	521^bc^	98^cd^	187	37
	2	9.1^bc^	339^de^	110^cd^	140	59
	5	10.4^b^	275^e^	87^cd^	148	67
	250	28.2^a^	179^f^	81^cd^	78	79
Control		7.5^cd^	7.5^cd^	355^a^	300	0

Means in the same column followed by a different letter(s) are significantly different at *p* < 0.05 according to Duncan’s multiple range tests.

The number of egg masses with all concentrations tested generally showed significant reductions in comparison with the control. The 0.25, 1, 2, and 5 ppm applications negatively affected egg numbers per egg mass, especially with fenamiphos and oxamyl. In the control, the number of eggs/egg mass was 300, while with the 1 ppm fenamiphos or oxamyl the number of eggs/egg mass was reduced to 176 and 187, respectively, and their lowest values were 90 and 78 respectively, with 250 ppm.

### Effects of sub-lethal concentrations of nematicides on *M. incognita* host finding ability

**Figure 3:**
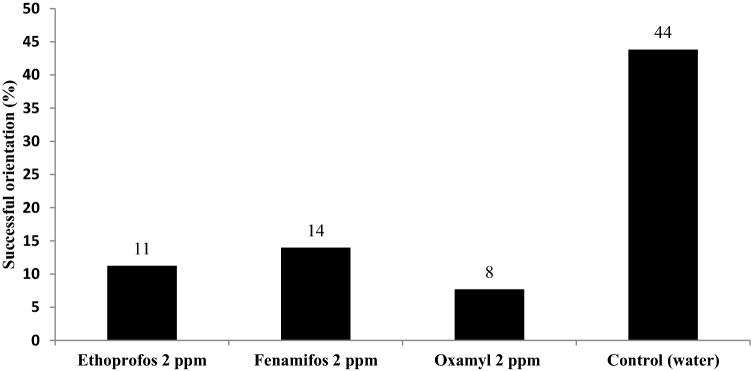
Percentages of *M. incognita* juveniles that were successfully located to their host roots after exposure to 2 ppm of the three nematicides.

The effect of one hour of dipping in 2 mg/L of each of the three different nematicides on the orientation behavior of the *M. incognita* juveniles toward the host roots is shown in Table 2 and Fig. 3. For the control, 44% of the juveniles were found to successfully orientate toward the roots, which represented the highest ratio. However, dipping the *M. incognita* larvae in 2 mg/L ethoprophos, fenamiphos, or oxamyl greatly reduced this ratio, as they resulted in 11%, 14%, and 8% successful orientations, respectively. In contrast, 13%, 0%, 6%, and 8% of the larvae were disoriented in the non-seedling bulb (containing sand only) with the ethoprophos, fenamiphos, oxamyl, and water control, respectively. In the middle barrel (the area where the juveniles were injected), the number of nematode larvae was found to be higher with all nematicide treatments after 72 h, as only 48% of the juveniles did not move away from the middle barrel within the mentioned period. However, this increased to 86% for both the fenamiphos and oxamyl and 76% for the ethoprophos.

**Table 2:** Effect of low nematicide concentrations on the orientation of *Meloidogyne incognita* juveniles to host roots after 72 h.

Treatment (2 ppm)	Total^1^ injected J2	Retrieved^2^ J2	Attracted Juveniles					
			Seedling bulb		Middle barrel		Non-seedling bulb	
			No	%	No	%	No	%
Ethoprophos	800	605	68	11	458	76	80	13
Fenamiphos	800	616	85	14	531	86	0	0
Oxamyl	800	489	38	8	422	86	30	6
Control	800	508	223	44	245	48	40	8

^1^Is the total number of injected Juveniles at 0 times, and ^2^is the total number of retrieved Juveniles after 72 h.

### AChE activity and ATPase total activity

The specific activities of the AChE in *M. incognita* were 415.3, 339.9, 505.6, 470.0, 468.6, and 348.7 nM ATChI/min/mg protein for oxamyl, ethoprophos, and fenamiphos at concentrations of 5 and 250 mg/L, respectively, while the value was 535.7 nM ATChI/min/mg protein in the *M. incognita* control group (Table 3).

**Table 3:** AChE (nmol/min/mg protein) and ATPase (nmol Pi/mg protein/15 min) specific activity and the inhibition (%) of *Meloidogyne incognita* with the oxamyl, ethoprophos, and fenamiphos treatments after 48 h post-treatment.

Treatments	Conc. (mg/L)	AChE specific activity (nmol /min/mg protein) ± SE	Inhibition (%)	ATPase-specific activity (nmol Pi/mg protein/ 15 min) ± SE	Inhibition (%)
Control	–	536 ± 7.58^a^	–	6.59 ± 1.63^a,b^	–
Oxamyl	5	415 ± 12.0^a,b^	22.5	4.27 ± 1.50^b,c^	35.2
	250	340 ± 33.7^b^	36.5	7.27 ± 0.54^a^	–
Ethoprophos	5	505 ± 50.3^a^	5.61	1.16 ± 0.22^d^	82.4
	250	470 ± 21.1^a^	12.3	1.65 ± 0.22^c,d^	74.9
Fenamiphos	5	469 ± 67.6^a^	12.5	1.13 ± 0.33^d^	82.9
	250	349 ± 23.8^b^	34.9	0.67 ± 0.12^d^	89.8

Each value is the mean ± SE of 3 replicates, means within the same column followed by the same letter are not significantly different (*P* ≤ 0.05; Duncan’s multiple range test).

The order of inhibition potency for the tested compounds against *M. incognita* AChE was: oxamyl (250 mg/L) > fenamiphos (250 mg/L) > oxamyl (5 mg/L) > fenamiphos (5 mg/L) > ethoprophos (250 mg/L) > ethoprophos (5 mg/L), and the inhibition percentage values were 36.5%, 34.9%, 22.5%, 12.5%, 12.3%, and 5.61%, respectively (Table 3).

The total specific activities for the ATPase in *M. incognita* were 4.27, 7.27, 1.16, 1.65, 0.76, and 1.13 nM Pi/mg protein/15 min for oxamyl, ethoprophos, and fenamiphos at concentrations of 5 and 250 mg/L, respectively, compared to 6.59 nM Pi/mg protein/15 min in the *M. incognita* control group (Table 3).

The order of inhibition potency for the tested compounds against *M. incognita* ATPase was: fenamiphos (250 mg/L) > fenamiphos (5 mg/L) > ethoprophos (5 mg/L) > ethoprophos (250 mg/L) > oxamyl (5 mg/L), and the inhibition percentages values were 89.8%, 82.9%, 82.4%, 74.9%, and 35.2%, respectively. When compared to the control, 250 mg/L oxamyl was not found to significantly increase the ATPase activity (Table 3).

## Discussion

Plant-parasitic nematodes are effectively suppressed by chemical nematicides^32^. The recommended doses, however, are associated with toxicity concerns and negative effects on non-target organisms. In nematode management, using nematicides at lower doses may not only alleviate such negative concerns, but will also reduce crop production costs.

The in vitro experimental data showed that all of the tested nematicides at 250 ppm were highly toxic, resulting in 80–90% juvenile mortality. In general, the three tested nematicides did not show promising toxic effects at lower concentrations (0.25, 1, 2, and 5 ppm), except for ethoprophos at 5 ppm, which caused 58% juvenile mortality.

The pot experiment results showed that the highest concentration (250 ppm) achieved the highest significant negative effect on *M. incognita* gall numbers and egg masses, which generally reached up to 79 and 85% reduction in gall numbers for oxamyl and fenamiphos, respectively. Moreover, the lower tested concentrations (from 0.25 to 5 ppm) also resulted in significant reductions in these parameters. These reductions generally ranged from 30% and up to 67%. Such suppression in the *Meloidogyne* incognita infection at these low concentrations cannot be attributed to in vitro juvenile mortality, particularly at 0.25 and 1 ppm concentrations, which did not exceed 7% juvenile mortality with any of the nematicides investigated. Reduced nematode infection at low concentrations may be due to their inability to orient towards the host root. To further investigate this assumption, we performed a chemotaxis assay.

In the chemotaxis assay, the orientation of *M. incognita* infective juveniles toward susceptible host roots was measured 72 h after exposure to the 2-ppm concentration of ethoprophos, fenamiphos, and oxamyl. The results revealed that a high percentage (76 -86%) of the nematode population were unable to move towards the host roots and even did not leave the injection point area (the middle barrel) with all of the three nematicides, recording the highest ratios of the intact juveniles in case of fenamiphos and oxamyl (86% of the population for both) when compared to the control (48%). According to these findings, nematode juveniles had a physiological impairment that hindered their ability to infect plant roots, which may explain the reduction in nematode infection in the pot experiment under sublethal nematicides concentrations.

In our study, AChE specific activity evaluation results showed that the low concentrations of ethoprophos, fenamiphos, and oxamyl caused slight non-significant inhibition in AChE of *M. incognita*, these inhibition percentages were 5.61, 12.5 and 22.5, respectively. Besides, ethoprophos with its high and low concentrations did not show AChE inhibition effects. Regarding ethoprophos which belongs to the phosphates with a sulphur moiety in the phosphoryl head , these results coming along with Al-Rehiayani (2008), who found that such chemical group had poor activity against nematode AChE inhibition.

ATPase specific activity evaluation showed that the low concentrations (5 ppm) of ethoprophos, fenamiphos and oxamyl strongly inhibited ATPase specific activity by 82.4, 82.9 and 35.2% respectively. This inhibition of ATPase activity could explain the negative effects on *M. incognita* infection and fecundity observed in the pot experiment when treated with sublethal concentrations of these nematicides. In the free living nematode, *Caenorhabditis elegans*, ATPase inhibition also caused negative effects on the nematode development^34^ and shortened lifespan, reduced body size and retarded postembryonic development^35^.

Finally, the examined nematicides, which are carbamate and organophosphate compounds, have been previously identified as AChE inhibitors. The results, however, allude to their stronger inhibitory effects on ATPase. This was especially noticeable with ethoprophos, which possesses a sulphur moiety that prevents it from influencing AChE. Although the physiological abnormalities caused by low nematicide concentrations did not kill *M. incognita* J2, the negative effects on nematode orientation to the host, infection, and fecundity were clear.

## Conclusion

The application of low concentrations of the nematicides is a promising tactic to manage *M. incognita* in pots. Field trials, however, are required to assess the efficacy of this strategy in the open field. The tested concentrations in our study were hundreds of times lower than the recommended doses. Managing nematodes by application of such low concentrations will greatly reduce toxicity issues for humans and the ecosystem while also being cost-effective.

## Data Availability

The datasets generated during and/or analyzed during the current study are available from the corresponding author on request.
